# α-Catenin Localization and Sarcomere Self-Organization on N-Cadherin Adhesive Patterns Are Myocyte Contractility Driven

**DOI:** 10.1371/journal.pone.0047592

**Published:** 2012-10-15

**Authors:** Anant Chopra, Akash Patel, Adrian C. Shieh, Paul A. Janmey, J. Yasha Kresh

**Affiliations:** 1 Department of Cardiothoracic Surgery, Drexel University College of Medicine, Philadelphia, Pennsylvania, United States of America; 2 School of Biomedical Engineering, Science and Health Systems, Drexel University, Philadelphia, Pennsylvania, United States of America; 3 Institute for Medicine and Engineering, University of Pennsylvania, Philadelphia, Pennsylvania, United States of America; University of California, San Diego, United States of America

## Abstract

The N-cadherin (N-cad) complex plays a crucial role in cardiac cell structure and function. Cadherins are adhesion proteins linking adjacent cardiac cells and, like integrin adhesions, are sensitive to force transmission. Forces through these adhesions are capable of eliciting structural and functional changes in myocytes. Compared to integrins, the mechanisms of force transduction through cadherins are less explored. α-catenin is a major component of the cadherin-catenin complex, thought to provide a link to the cell actin cytoskeleton. Using N-cad micropatterned substrates in an adhesion constrainment model, the results from this study show that α-catenin localizes to regions of highest internal stress in myocytes. This localization suggests that α-catenin acts as an adaptor protein associated with the cadherin mechanosensory apparatus, which is distinct from mechanosensing through integrins. Myosin inhibition in cells bound by integrins to fibronectin-coated patterns disrupts myofibiril organization, whereas on N-cad coated patterns, myosin inhibition leads to better organized myofibrils. This result indicates that the two adhesion systems provide independent mechanisms for regulating myocyte structural organization.

## Introduction

The synchronized beating of the heart is critical to its proper function and is made possible by the intimate electrical and mechanical coupling between individual cardiac myocytes. Adherens junctions (AJs) comprised of N-cad provide not only mechanical connections between myocytes in order to maintain the tensional integrity and alignment of their cytoskeletons but are also an important part of the myocyte mechanosensory apparatus [1], [2].

Transmission of mechanical forces through connections between integrins and the extracellular matrix (ECM) in cardiac myocytes and other cell types has been associated with a plethora of cytoskeletal adaptor proteins such as talin, filamin, vinculin, and paxillin, and signaling proteins like focal adhesion kinase and the small GTPase RhoA; these proteins and others control formation of the focal adhesion complex (FA) (for review see) [3], [4], [5]. FA's relay biochemical signals to the cytoskeleton and the nucleus that are necessary for cell remodeling. In contrast to the detailed molecular description of FA formation and signaling, the adaptor proteins relaying biochemical signals initiated by forces transmitted through cadherin adhesions are less well established. AJs are stabilized by the dynamic binding of the intracellular N-cad domain to the actin cytoskeleton via molecular complexes consisting of β-catenins [6], α-catenins [7], p120, and related molecular partners. α-catenin is thought to be the bridge between the N-cad/β-catenin complex and the F-actin cytoskeleton. We chose to study α-catenin since it has been shown to alter its expression and conformation relative to cell-cell adhesion stability and force transmission in epithelial cell lineages [8], [9]. This paper explores the possible role of α-catenin in the mechanosignaling complex of N-cadherin mediated adhesions in cardiac myocytes.

The use of single cell micro-patterns provides an effective way to control a cell's shape and mechanical microenvironment in a reproducible manner. “Y” shaped micropatterns, in particular, provide a unique geometry with three polarized anisotropic ends and an isotropic center [10]. Higher forces have been calculated and greater concentrations of focal adhesion proteins like vinculin are localized at the apices (corners) of spatially confined muscle cells on constrained patterns [11], [12]. A similar phenomenon has been described in other studies examining non-muscle cells suggesting a general mechanism of constrainment-driven actin assembly and force distribution [10], [13], [14], [15]. Computational models for estimating forces by acto-myosin generated contractility in single cells on this geometry have demonstrated that the highest internal stresses exerted within these cells occur at the apices of the “Y” shaped geometry, whereas the center of these cells generates little internal stress [16]. We and others have shown that self assembly of stress fibers [10] and cardiac sarcomeres [2], [11] are a function of adhesion geometry constraints. Therefore, cells on “Y” shaped micro-pattern geometries provide an ideal model system to study the effects of internal stress gradients on cell structure and protein distribution. Using this system, we tested the hypothesis that α-catenin alters its subcellular localization in response to variations in stress found at the N-cadherin adhesion complex.

The use of a standardized single cell approach to understand cytoskeletal organization and protein mechanosensing can give insight into the molecular players involved in cell-cell adhesion dynamics. Additionally, it provides an opportunity to monitor the sub-cellular localization of responsive proteins under well-defined mechanical stress gradients, which would be difficult to control in a normal cell-cell pair or confluent culture model. It has been shown that geometrical constrainment of contractile cells results in controlled focusing of forces, where peak stresses were confined to the apices of concave patterned shapes and were associated with actin cables polarized to these points [11], [13]. These results provide insight into the mechanism underlying force sensing through cell-cell junctions required to maintain cardiac tissue tensional homeostasis.

## Results

### Sarcomere organization depends on ligand type

**Figure 1:**
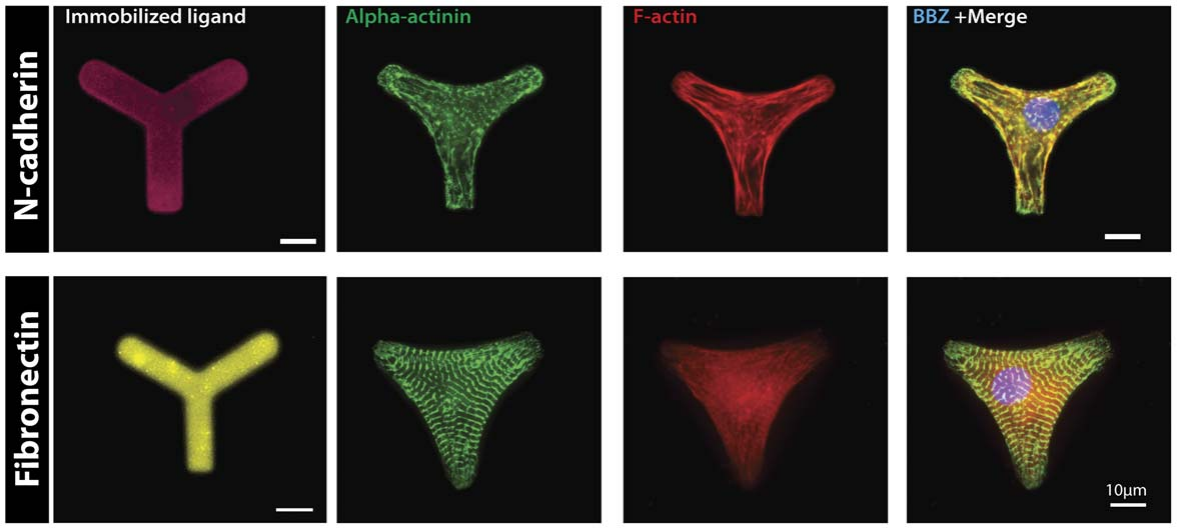
Myocytes plated on ‘Y’ patterns for 72 hours (Top row, left to right) N-cad stained micropattern, myocytes on N-cad coated patterns stained for α-actinin (green), F-actin (red), merged image of cardiac myocyte (nucleus stained with Bisbenzimide (BBZ), blue) show poor α-actinin and thin filament organization. (Bottom row, left to right) Fibronectin stained micropattern, myocytes stained for the same cytoskeletal markers show highly organized sarcomere registration governed by the anisotropy of the adhesion geometry. Scale bar indicate 10 µm.

Neonatal ventricular myocytes were cultured on N-cad coated Y patterns (Fig.1) for a period of 72 hours to allow for sufficient maturation of N-cad mediated contacts and complete pattern occupation. As a control, cells were plated on fibronectin (Fn)-coated Y patterns (Fig.1) for the same period of time. Cardiac myocytes on N-cadherin micropatterns (Fig. 1, upper row) demonstrate diffuse sarcomeric disorganization. In contrast, on Fn micropatterns (Fig. 1, bottom row) myocytes show well-organized sarcomeric striations and distinct z-lines. Noteworthy is the fact that sarcomeres can assemble over the non-adherent edges of the cell, preserving its continuum and demonstrating the unique structural lattice properties of the myocyte cytoskeleton. The polarity of the myofibrils on Fn coated patterns and F-actin bundles on N-cad was dictated by the anisotropy generated by the apices of the cell.

### Sarcomere organization is actomyosin contractility dependent

It is postulated that the myosin-II-tensed cytoskeleton leads to structural anisotropy of the stress fibers and thin filaments within the cell. The dynamic remodeling of the actin cytoskeleton, as demonstrated by *in silico* modeling for square cells positioned on four adherent posts, inevitably leads to the dynamic transverse compression-driven compaction of stress-fibers [17]. To test whether myosin-generated tension is responsible for the compaction and alignment of myofibrils on Fn- or N-Cad coated patterns, we next inhibited myosin activity.

**Figure 2:**
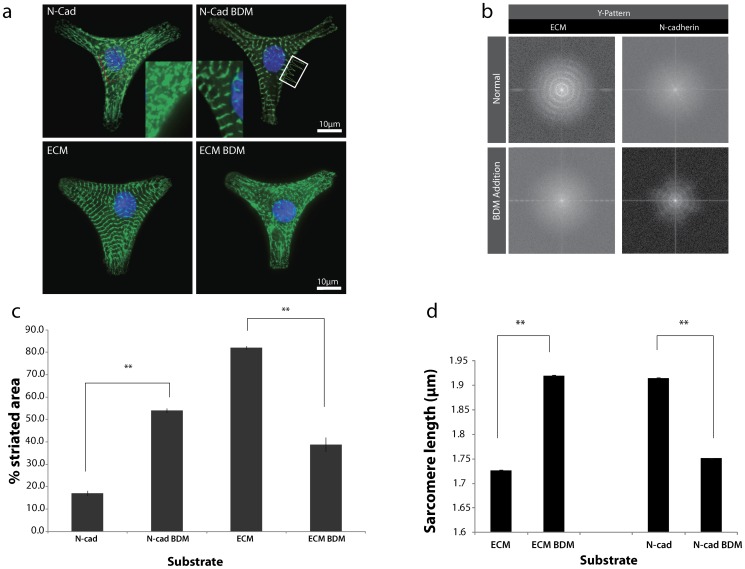
Cardiac myocyte myofibrillar assembly is sensitive to ligand type and contractility. (a) Representative image of myocytes on N-cad and Fn stained for α-actinin (green) demonstrating the appearance of sarcomeres after BDM treatment (inset – zoomed region of the same cells) whereas the opposite effect is observed on Fn. (b) Fourier transform spectra of single myocytes cultured for 72 hours on N-cad and Fn coated micropatterns before and after BDM treatment. α-actinin staining was used to identify repeating patterns/striated myofibrils. Myocytes on N-cad did not show patterned Fourier transform spectra when compared to Fn. However, the repeating patterns appear after BDM treatment on N-cad, whereas on Fn, they are lost. Myocytes patterned on rectangular Fn coated substrates show a uniaxial Fourier spectrum compared to the circular repeated patterns on ‘Y’ shapes indicative of tri-axial anisotropy. (c,d) Quantitative collective measurements of % striated area and sarcomere length (in µm) as a function of substrate composition with and without the addition of BDM show a similar trend. **p<0.01, error bars ±1 SEM for 20 cells

Inhibition of contractility with 2,3-butanedione monoxime (BDM), a myosin ATPase inhibitor, on fibronectin micropatterns resulted in heterogeneous sarcomere organization as shown by scattered or punctate α-actinin staining, higher number of misaligned z-lines, increased effective sarcomere length, and loss of the striated pattern, indicating that myosin contractility is required for myofibril compaction and organization on these substrates (Fig. 2 a,b,c &d). In contrast to what was seen on fibronectin, myosin inhibition in myocytes on N-cad coated patterns leads to better organized myofibrils with aligned z lines, giving rise to normally registered striation (Fig. 2 a,b,c). The sarcomere length decreased from ∼1.9 µm to the normal sarcomere length of ∼1.7 µm, opposite to what was seen on fibronectin patterns (Fig.2 a,d). These results indicate that the regulation of sarcomere assembly is contractility dependent but is differentially responsive to the cell-cell and cell-ECM adhesion in terms of its cytoskeletal structure.

### Radius of curvature as a measure of contractility on N-cadherin coated Y patterns

The edges of cells on “Y” shaped micro-patterns demonstrate varying curvatures depending on the contractile strength of the cell [18]. The measure of the resultant concavity and/or convexity, in terms of curvature radius, can be used to determine the contractile strength of the cell [10], [18]. In this model construct, the radius of curvature (R) was shown to be proportional to line tension (force parallel to side of cell) (λ) divided by surface tension (perpendicular force to side of cell) (σ): R =  λ/σ [18]. The radius of curvature was confined to the resting mechanical state of myocytes. The variability from diastole and systole (Fig.S1) is relatively small since the cells are confined to pseudo-isometric contraction when plated on stiff glass substrates. For this study, measures of static contractility are more significant since they are more closely related to protein localization and overall cytoskeletal structural changes. Transient changes in the radius curvature during a beat cycle did not affect our assay because we were measuring the difference in the pre- and post- myosin inhibition states.

**Figure 3:**
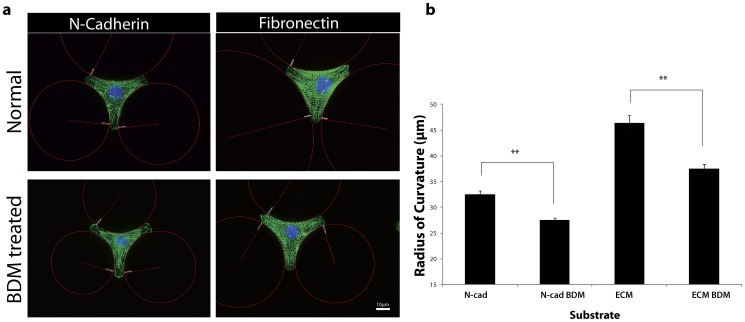
Radius of curvature is a function of contractility (a) Representative images of radius of curvature with and without BDM (b) Quantitative assessment shows that the radius of curvature decreases with myosin inhibition in myocytes on both N-cad and Fn substrates. **p<0.01, error bars ±1 SEM for 40 cells.

The radius of curvature was measured for myocytes on N-cad and Fn (ECM) coated substrates (Fig. 3). The addition of BDM resulted in a smaller radius of curvature for myocytes cultured on Fn and N-cad indicative of a decrease in contractility relative to their pre-BDM states (p<0.01) (Fig 3. b). This result shows that the radius of curvature is an acceptable readout of the relative cardiac myocyte contractile state at a whole cell level.

### α-catenin localizes to areas of high internal stress on N-cad coated substrates

**Figure 4:**
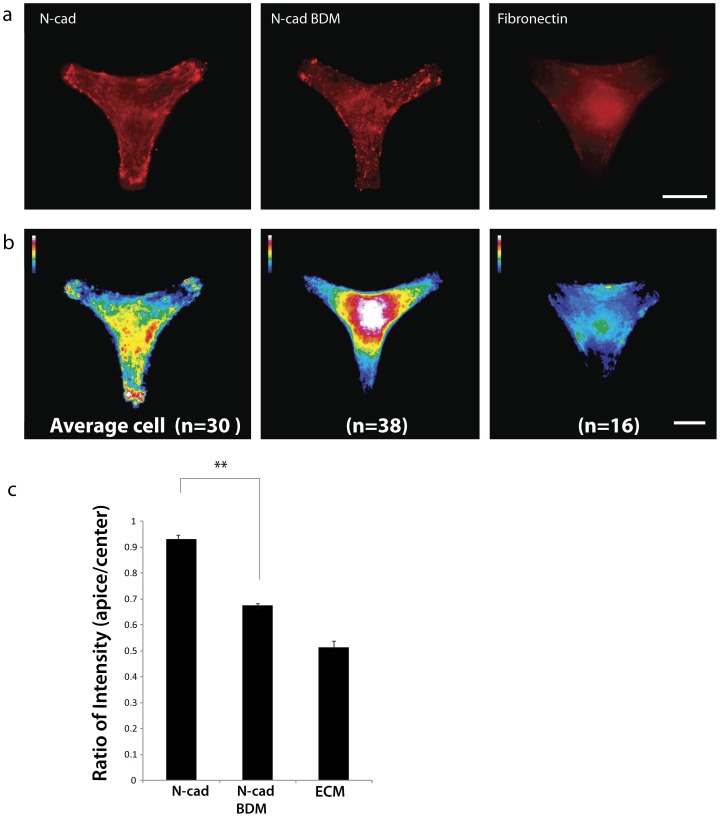
α-catenin localization is enhanced at the apices. (a)(Left to right) Representative images of cardiac myocytes stained for α-catenin (red) on N-cad patterns with and without BDM compared to Fn control; (b) (Left) Average cell of α-catenin stained cardiac myocytes on N-cadherin substrate (n = 30 cells) shows localization at the apices, (center) N-cadherin substrate after addition of BDM, a myosin inhibitor (n = 38 cells), note the absence of α-catenin localization at the apices and preferential localization to the perinuclear region, (right) Fn used as a control shows no preferential localization to the apices (white =  most intense staining, blue = least intense staining); (c) Ratio of intensity of α-catenin staining on the apices of the Y-shaped micropattern to the intensity of α-catenin staining in the center of the micropatterns shows enhanced localization at the cell apices when contractility is not inhibited. **p<0.01, error bars  =  ±1 SEM. Scale bar indicates 20 µm.

Myocytes on N-cad and Fn coated patterns were stained for α-catenin, and an average ‘standardized’ cell was generated by superimposing images of cardiac myocytes using ImageJ to visualize and quantify its localization (Fig. 4 a,b). The apices of cardiac myocytes on Y-shaped patterns represent areas of highest internal stress. Our results show that there is clear localization of alpha-catenin to the apices of N-cad coated Y-shaped micropatterns (Fig. 4 a,b). Addition of BDM decreased cell contractility and reduced the localization of α-catenin to the apices (Fig.4 b). This effect was also demonstrated quantitatively by an intensity heat map (Fig. 4 c). There was a significant difference (p<0.01) between the intensity of α-catenin fluorescence at the apices of the Y-pattern versus the center. No apical specific localization was observed on the control Fn coated patterns.

Localization of α-catenin was sensitive to physical cues at even smaller length scales than those of the cell apex relative to the center. The patterns used in this study were approximately symmetric to rotation around 120 degrees, but one of the 3 arms was systematically more squared off compared to the other two arms, which were rounded. Interestingly, there was increased α-catenin localization to the squared apex of the Y-pattern relative to the other apices. Sharp edges are known to be areas of especially larger internal stresses in other systems[19], [20]. These results indicate that α-catenin localization is contractility dependent and specific to N-cad. The localization of α-catenin to regions of highest internal stress is similar to that observed for vinculin on Fn [10], [15] (data not shown) suggesting that α-catenin plays a similarly important role in force transduction via cadherin mediated adhesions in cardiac myocytes.

## Discussion

As shown by both in vivo and in vitro culture experiments, the formation and maintenance of cadherin adhesions are crucial for myofibrillar assembly and alignment [21], [22]. In addition, cadherin mediated adhesions provide the needed mechanical stability to maintain proper gap junction electrical communication in cardiac myocytes [23], [24]. Recent work has clearly established cadherins as part of the cell-cell mechanosensory complex capable of exerting and responding to forces [1], [25], [26]. We have previously shown that mechanical stimuli from cadherin adhesions can regulate myocyte structure and function [1]. What is lacking is the elucidation of the signaling cascade downstream of N-cadherin involved in communicating the mechanical stimuli that direct myocyte structuring. Therefore a model of adherens junction-controlled cytoskeleton regulation in which the N-cadherin functionalized two dimensional (2D) surface served as a surrogate cell-cell contact was used with the intended purpose of modulating experienced stress fields to elucidate the behavior of the signaling proteins involved.

The exact nature of α-catenin binding to the N-cad/β-catenin complex and the F-actin cytoskeleton has been a much debated topic [27]. This study clearly shows that α-catenin spatial localization is sensitive to changes in contractility and boundary conditions dictated by N-cad topography. The stoichiometry of α-catenin's dynamic binding to the N-cad/β-catenin complex can be regulated by many factors [28]. The simplest explanation of our results could be that one of the factors enhancing the dynamic binding of α-catenin to the N-cad complex is the degree of internal stress at the cell-cell junction. The spatial organization of adhesion complexes is sensitive to geometric cues and boundary conditions (corners) and may be responsible for the observed anisotropy of the myofibrillar structures. Cytoplasmic pools of α-catenin can assume a homodimeric complex, regulating F-actin bundle assembly and lamellipodial activity at the cell-cell junction [29], [30], [31]. It can be postulated that the enhanced α-catenin localization to the apices could be a result of increased lamellipodial activity in response to high stresses at the apices through N-cad adhesions, by analogy with cells cultured on Fn coated micropatterns that show enhanced lamellipodial dynamics at the apices [15]. One can infer from the results presented in this paper that these α-catenin pools form as a direct result of force gradients generated at cell-cell junctions and allow further F-actin bundling locally. However, further studies will be required to show the magnitude of these forces and the extent to which α-catenin in these areas is homodimeric or in a heterodimeric complex with β-catenin. These results provide supporting evidence that the α-catenin/cadherin complex may serve as a key mechanosensory regulator of cardiac myocyte cytoskeletal structure. This single cell standardized model system can also be used to identify other potentially interesting mechanosensory candidates such as p120 [32].

In addition, our results demonstrate that there is a different set point for sarcomeric organization and remodeling depending on whether adhesion and force transmission is mediated by cadherins or integrins. Lower myosin activity is preferential for cadherin dependent assembly and preservation of sarcomeres. This condition has been observed and quantified previously for myocytes on N-cad coated soft and stiff polyacrylamide surfaces [1]. Higher striated areas were noted for myocytes with less contractility coated on soft surfaces as compared to myocytes with higher contractility coated on stiff substrates [1]. Myosin does not localize to the cell-cell junction; nonetheless myofibrils assembled, organized, and were preserved in other regions of the cardiac myocyte [33]. Cadherin- and integrin-based junctions can sustain stresses of similar magnitudes [1], [34], but the nature of subsequent cytoskeletal remodeling is different due to the specific involvement of proteins and their interaction in assembling and orienting F-actin bundles in response to these stresses. Normal myofibrillar assembly is driven by actomyosin forces and F-actin reorganization [35]. Therefore, its assembly and remodeling is expected to be responsive to the nature of load resistance mediated by specific ligands. In support of this line of thinking, stem cells show similar changes in cytoskeletal properties when subjected to cyclic forces from integrin and cadherin adhesions and respond differently in terms of spreading and differentiation [36].In summary, our results indicate that cell-cell and cell-ECM adhesion systems provide independent degrees of freedom for tuning cardiac myocyte structural organization.

## Materials and Methods

### Myocyte isolation, plating and culture

Neonatal ventricular rat myocytes (NVRM) were harvested from the hearts of 1- to 3-day-old euthanized Sprague-Dawley rat pups using a cell isolation kit (Cellutron Life Technology, Baltimore, MD) as described previously [1]. Briefly, cells were isolated from the muscle tissue of the two ventricles. The cardiac myocytes were pre-plated for 1 to 2 h to purify the myocyte population. The cells were cultured at a density of 7,000 cells/cm^2^ in high serum (10% fetal bovine serum) medium (Cellutron) on the various gel substrates for 24 h at 5% CO_2_ and 37°C. The medium was changed to low serum (2% fetal bovine serum) and maintained for another 48 h. This time period proved sufficient to allow the cells to attach completely to the micropatterns.

### Myocyte micropatterning

‘Y’ shaped glass micropatterns of total area 1100 µm^2^ (dimensions: distance from the center to tip of a branch is 25.8 µm, the line width is 8 µm) (CYTOO, Grenoble, France) were either purchased pre-coated with fibronectin or coated later with N-cad as described previously [1]. Briefly, pre-activated micropatterned glass substrates were coated with anti-Fc (Jackson ImmunoResearch Laboratories) at a concentration of 2.5 µg/cm^2^ for 4 hours, according to company instructions. Anti-Fc coated substrates were then incubated with recombinant human N-cad Fc chimera (R&D Systems) at a saturating concentration of 5 µg/cm^2^, for a minimum of 2 h. To visualize uniform coating of N-cad, the micropatterns were immuno-stained with anti-rabbit-N-cad antibody (Santa Cruz).

### Morphology of cells on N-cad and Fn coated micropatterns

To visualize myocyte cytoskeleton and α-catenin, cells were fixed with 4% paraformaldehyde (Sigma) for 10 min and then washed with 1× PBS1xPBS. Fixed gel substrates were permeabilized with 0.1% Triton X-100 in TBS for 10 min and washed 3 times for 5 min in TBS. Following permeabilization substrates were then incubated for 1 h at room temperature with mouse monoclonal anti-α-actinin antibody to visualize sarcomere definition and assembly (1∶400; Sigma) and anti-α-catenin antibody (1∶200; Abcam). The substrates were washed 3 times with TBS before secondary antibody was applied. Secondary antibody, anti-mouse Alexa 488 (1∶500; Invitrogen), anti-rabbit CY5 (1∶500; Invitrogen) was prepared in TBS solution containing 1% BSA. Phalloidin–tetramethylrhodamine B isothiocyanate (1 µg/ml; Sigma) was used to visualize F-actin formation, and bisbenzimide (1 µg/ml; Sigma) to visualize the cell nucleus. Substrates were incubated in secondary antibody for 1 h and washed three times with TBS containing 0.1% Tween. The cells were visualized under a conventional microscope (Carl Zeiss, Thornwood, NY) at x20 and x63. Images were acquired using proprietary software (Axiovision; Carl Zeiss).

### Image analysis

Fast Fourier Transform was used to evaluate repeated striation patterns for representative cells on N-cad and Fn coated ‘Y’ patterns, using the image J software (National Institutes of Health, Bethesda, MD). The percentage of myocyte striation area was confined to areas displaying sarcomere lengths ranging from ∼1.7 to 1.8 µm normalized to the entire cell area. Sarcomere length was calculated by measuring the distance between peak intensity spikes across a random sampling of myofibrils. Radius of curvature was measured across all free cell edges using proprietary software (Axiovision; Carl Zeiss). To develop the “average cell”, i.e. the average spatial distribution of the α-catenin label, acquired images were aligned using the fluorescently labeled N-cad and Fn micropatterns and stacked using a custom ImageJ macro developed by CYTOO [10], [13]. The “average cell” was obtained by calculating the average intensity of each pixel over the stack using Image J software. To calculate the ratio of apex to center intensity, the average fluorescence intensity in each of the three apices of the “average cell” was divided by the average fluorescence intensity in the center of the “average cell”. The areas of interest used in the fluorescence intensity analysis were confined to 40 µm^2^ for the apices and 120 µm^2^ for the center respectively.

### Statistical analysis

Student t-test was performed to show a significant statistical variation for experiments (where *P*<0.05 was considered significant unless otherwise specified). Error bars indicate standard error unless otherwise specified.

## Supporting Information

Figure S1 **Comparison of two partial images showing changes in radius of curvature of myocytes on fibronectin coated Y-shaped patterns during active relaxation (diastole) and contraction (systole) phase.** The difference in radius of curvature were minimal <1 µm attributed to pseudo-isometric contraction on an especially stiff substrate (glass).
(TIF)
