# Supplementary figures and images for: α-Catenin Localization and Sarcomere Self-Organization on N-Cadherin Adhesive Patterns Are Myocyte Contractility Driven
Source: PLoS One. 2012 Oct 15;7(10):e47592. doi: 10.1371/journal.pone.0047592 (PMC3471892; doi:10.1371/journal.pone.0047592)

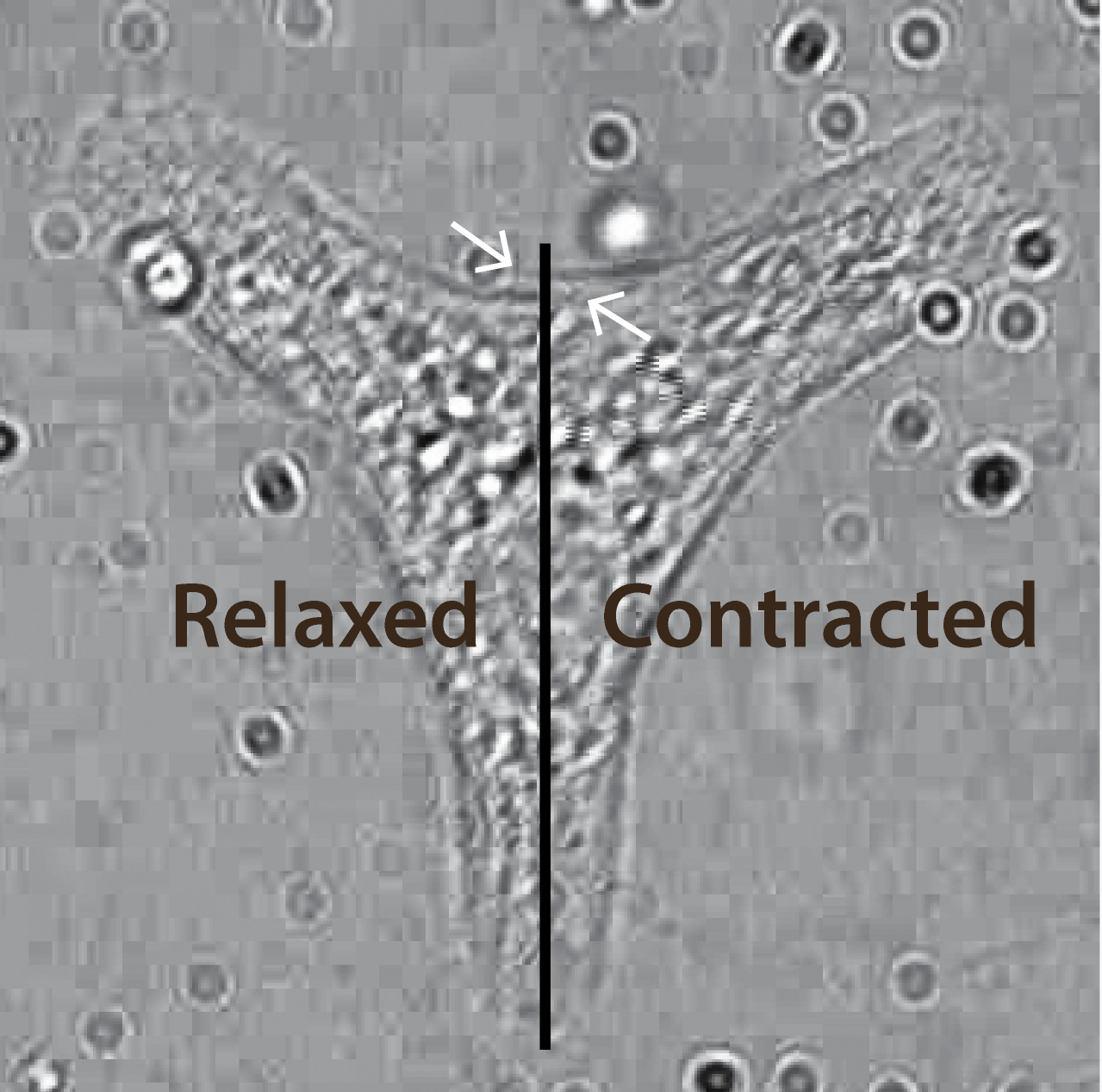

Supplement: Figure S1 — Comparison of two partial images showing changes in radius of curvature of myocytes on fibronectin coated Y-shaped patterns during active relaxation (diastole) and contraction (systole) phase. The difference in radius of curvature were minimal <1 µm attributed to pseudo-isometric contraction on an especially stiff substrate (glass). (TIF) [file pone.0047592.s001.tif]
